# Elements of General and Pathological Anatomy

**Published:** 1851-10

**Authors:** 


*Elements of General and Pathological Anatomy, presenting a view of the present state of knowledge in three Branches of Science.* By DAVID CRAIGE, M. D., F. R. S. E. &c., &c. Second edition, enlarged, revised, and improved. PHILADELPHIA: LINDSAY & BLAKISTON, 8vo, pp. 1072.

THE above is the title of a recent work in its second edition, which gives most of the facts acknowledged by modern pathological anatomists.

It examines most of the prominent opinions of almost all writers upon this subject, and must be valued principally as a comprehensive review of the thoughts and views of others. For this reason it will be esteemed a good work for reference, and as its nomenclature is full even to a fault, there will be found names for all diseases in abundance. The vast accumulation of materials for such a work, spread through such an immense amount of matter, renders such an undertaking a labor of unusual toil. The author has done well as far as he has gone, and serviceably condensed a vast amount of knowledge in a small space so that, for the student, or the physician's library, this must be esteemed a valuable acquisition.

We conceive it a great error in such a work, to devote so small a portion, and in so careless a manner to the subject of the *Teeth,* and which is too often the case in almost all works of the kind. This culpable want of attention, to so large and highly important a branch, both of anatomy and pathology, gives rise to the prevailing deficiency of correct imformation upon the growth, structure, and diseases of the teeth existing in the medical profession, and that, too, in high places. Mr. Craige, in this particular, is no better than others who have preceded him, and we condemn such carelessness. A work proposing to give facts in anatomy and pathology, should embody all principal facts, and give such facts their true weight and importance. This work is wanting altogether in the minute structure of the teeth, &c., the mere pronouncing "that their development and growth is a process of much interest," is not sufficient for a scientific treatise. Again, on page 490 is found, in treating on the diseases of these organs, "that the mostfrequent cause of disease of the teeth, is inflammation of their internal pulp, an error too palpable to comment upon. Following, is another mistake, which the fault of carelessness will hardly cover. He says, "This, speaking from the previous quotation, by progressively destroying the membrane, impairs the nutrition of the tooth, which becomes carious in the bony pith, while the enamel cracks, and is cast off in the form of concave scales or crusts." All which is wrong, and contrary to facts, which any one at all acquainted with the subject will perceive to be glaring and culpable errors.

Inflammation of the pulp is always the effect of morbid or mechanical action from without, and never occurs only as a consequence of disease, as a result of a change in the normal condition of surrounding parts, or general system, and is never the prime cause of disease of the teeth. We regret that space prevents longer comment; and will finish, by stating, that in excepting a few doubtful points, the work is valuable in many respects, but offers traces of carelessness that should not occur in any future compilation.

